# Sociodemographic characteristics, complications requiring hospital admission and causes of in-hospital death in patients with liver cirrhosis admitted at a district hospital in Ghana

**DOI:** 10.1371/journal.pone.0253759

**Published:** 2021-06-24

**Authors:** Amoako Duah, Adwoa Agyei-Nkansah, Foster Osei-Poku, Francisca Duah, Bright Peprah Addo

**Affiliations:** 1 Department of Medicine, University of Ghana Medical Centre, Accra, Ghana; 2 Department of Medicine, University of Ghana Medical School, Korle-Bu Teaching Hospital, Accra, Ghana; 3 Laboratory Department, Ga-North District Hospital, Ofankor, Ghana; 4 Department of Biostatistics, St. Dominic Hospital, Akwatia, Ghana; Centre de Recherche en Cancerologie de Lyon, FRANCE

## Abstract

**Background:**

Chronic liver diseases including liver cirrhosis are a major cause of morbidity and mortality globally. Despite the high burden of liver cirrhosis in Ghana, data on this disease is lacking.

**Objective:**

To determine the sociodemographic characteristics, reasons for admission, and in-hospital mortality of patients with cirrhosis of the liver seen at a district hospital in Ghana.

**Methods:**

A prospective study was conducted involving one hundred and eighty-six (186) patients admitted on the medical wards in St. Dominic hospital with liver cirrhosis from 1st January 2018 to 24th June 2020. The patient’s demographic and clinical features were documented using a standardized questionnaire. Diagnostic biochemical and haematological tests as well as abdominal ultrasound scans were performed for all patients. They were followed up until death or discharge from hospital.

**Results:**

One hundred and eighty-six patients (186) with a median age of 46 years were included in the study. HBV was the main etiology of liver cirrhosis (38.7%) followed closely by alcohol consumption (38.3%). In-hospital mortality was 41.3% and the most frequent cause of death was hepatic encephalopathy (68.4%). The following were associated with death; Jaundice, weight loss, elevated bilirubin, international normalized ratio (INR), creatinine, blood urea nitrogen(BUN), Child-Pugh score, model for end-stage liver disease sodium score (MELDNa), and low sodium. However, hepatic encephalopathy, MELDNa, INR and BUN were independent predictors of in-hospital mortality on logistic regression analysis.

**Conclusions:**

In-hospital mortality in cirrhotic patients was high with the leading cause of death being hepatic encephalopathy. Timely diagnosis and adequate management of hepatic encephalopathy are necessary to prevent death from liver cirrhosis.

## Introduction

Chronic liver diseases (CLD) are a major cause of morbidity and mortality globally including Ghana [1]. Cirrhosis is the end-stage of all chronic liver diseases and is characterized by progressive fibrosis, scarring, and formation of regenerative nodules resulting in distortion of the normal liver architecture [2]. The burden of cirrhosis differs considerably across various geographical locations and according to sex, race, ethnicity, and socioeconomic level. There has also been a significant variation in the burden of cirrhosis over time. Mortality from liver cirrhosis increased worldwide from less than 899,000 in 1990 to over 1.32 million in 2017. In 2017, this constituted 2.4% of all deaths compared with 1.9% in 1990 [3]. Sub-Saharan Africa had the highest age-standardized mortality rate (32.2 deaths per population) according to the global burden of disease super-regions for 2017 [3]. From the global burden of disease study 2010, liver cirrhosis was the 13^th^ commonest cause of premature death in Ghana [4]. Chronic hepatitis B and C, excessive alcohol consumption and non-alcoholic fatty liver disease have been found to be the commonest causes of liver cirrhosis globally [5]. Chronic HBV infection is endemic in Ghana [6]. Despite being a worldwide health challenge with a major public health and economic burden, data assessing in-hospital mortality and morbidity due to cirrhosis are sparse, especially in many regions such as Africa [3]. In-hospital death in patients with liver cirrhosis and predictors of mortality are variable in various studies reported in literature. In a study conducted in Ethiopia by Terefe Tesfaye et al. [7], in-hospital mortality of cirrhotic patients was found to be 28.4% with hepatic encephalopathy and high bilirubin as predictors of death. This study, however, was limited by the small sample size and inability to perform advanced images like CT scan and MRI. It also did not have access to endoscopy to diagnose oesophageal varices. A similar retrospective study conducted in Morocco by Charif et al. [8], also found in-patients death of liver cirrhosis to be 8.7% with hepatic encephalopathy, hyponatremia, high creatinine, and leucocytes as predicting factors of death. Zubieta-Rodríguez et al. [9], reported 23.5% in-hospital mortality with high MELD score, leucocyte count and low albumin as independent factors related to death in Columbia and in-hospital mortality rate in a study conducted in Saudi Arabia was 35% with advanced age and MELD scores as predictors of death [10].

Data from Ghana on liver cirrhosis is scarce and therefore the gravity of liver cirrhosis is likely to be underestimated. Few studies have been carried out on liver diseases burden in Ghana. One of such studies reported cirrhosis as the commonest cause of death attributable to liver diseases in the country [11]. Moreover, existing studies are old and largely retrospective. There is a dearth of knowledge about in-hospital mortality of liver cirrhosis patients and the predictors of death in Ghana. This study aimed to assess the baseline characteristics, causes of hospital admissions, in-hospital mortality and predictors amongst patients with liver cirrhosis in a district hospital in Ghana.

## Methods

### Study design

This was a descriptive, observational prospective hospital-based study.

### Study area and period

The study was carried out at the Department of Medicine, St. Dominic Hospital (SDH) in Akwatia, Ghana from 1st January 2018 to 24th June 2020. SDH is a 339-bed district hospital located in the Denkyembour district, Akwatia in the Eastern region of Ghana and serves as the main referral center for surrounding district hospitals.

### Study population and sampling

The study population comprised adult patients with liver cirrhosis admitted to the medical unit of SDH during the study period who met the inclusion criteria. The sample size was determined using the Cochran formula for sample size calculation. With an estimated prevalence of 10.7% for in-hospital mortality of liver cirrhosis [12], a Z-score at 95% confidence level (1.96), and a level of significance of 0.05, the minimum sample size was calculated to be 148. Due to the high attrition rate for prospective studies, 186 participants were consecutively recruited after meeting study criteria and giving informed consent.

### Inclusion criteria

Patients with a diagnosis of liver cirrhosis (compensated or decompensated) who

1. Were 18 years and above.
2. Were admitted for the first time during the study period.

### Exclusion criteria

1. Patients with a previous history of HCC
2. Patient that were re-admitted within the study period

### Recruitment and follow up

Patients were recruited into the study on their index admission to SDH after meeting the inclusion criteria. Patients were followed up from this index admission until death or discharge. The primary outcome of interest was the cause of in-hospital mortality and the secondary outcome was predictors of in-hospital mortality.

### Data collection and measurements

#### Consenting patients who met the inclusion criteria were recruited

A questionnaire was administered to obtain information on socio-demographic data as well as relevant clinical history such as alcohol use. All patients underwent a thorough physical examination, and any stigmata of liver cirrhosis were documented. Further information collected included reasons for admission and the presence of cirrhosis-related complications such as ascites, variceal bleeding, spontaneous bacterial peritonitis (SBP) and other infections, hepatic encephalopathy and acute kidney injury including hepatorenal syndrome (HRS).

Fifteen (15mls) of venous blood sample was taken for hematological, biochemical, and serological investigations. Abdominal paracentesis was performed where indicated using aseptic technique and 30mls of ascitic fluid was taken for biochemistry and culture. The diagnosis of spontaneous bacterial peritonitis was based on the demonstration of more than 250 neutrophils/mm^3^ or positive fluid culture in ascitic fluid. Urine analysis (Proteins, leucocytes, erythrocytes, pus cell, and other urine abnormalities) was performed for all patients. Patients were screened for viral hepatitis B and C using a point of care rapid test kit for HBsAg and anti-HCV Ab to ascertain a viral etiology of liver cirrhosis. All the samples for laboratory analysis were taken within 24hrs of admission as well as the imaging studies.

Patients presenting with clinical features of upper gastrointestinal bleeding had an endoscopy. Furthermore, abdominal imaging using ultrasound scan and/or CT scan was performed for all patients to characterize the liver. The following details were noted: maximum vertical span of the liver; nodularity of liver surface; liver mass; spleen size (length of its longest axis); and presence of ascites. The Child-Pugh scoring (CPS) system and Model for End stage Liver Disease sodium (MELD-Na) score were calculated for all patients [13].

The diagnosis of liver cirrhosis was based on the presence of two or all three of the following criteria [14]:

1. Clinical signs of chronic liver disease (clubbing, palmar erythema, spider naevi, gynecomastia, distended abdominal veins, female pubic hair pattern, encephalopathy, splenomegaly, or ascites)
2. Impaired liver function test consistent with cirrhosis (elevated INR and low serum albumin)
3. Ultrasound diagnosis of cirrhosis (Shrunken or enlarged nodular liver with increased echotexture, a blunt edge, and distorted architecture, with or without a dilated portal vein, thickened gallbladder wall, splenomegaly, or ascites) And/or
4. APRI score of greater or equal to 2 [15].

A diagnosis of hepatic encephalopathy was made when the patient had impaired consciousness with a background liver cirrhosis in the absence of any neurological disorder or other causes of impaired consciousness [16]. The West Haven criteria were used in grading the encephalopathy [17]. Diagnosis of ascites was made based on the clinical features of abdominal distension, the presence of shifting dullness, and/ or positive fluid thrill. It was then confirmed by diagnostic paracentesis and/or an abdominal ultrasound scan [18].

Urinary tract infection was diagnosed in cases with a positive urine leucocyte and nitrite, and pus cell of >10 and/or positive culture of >10^5^ colonies/mL urine, with no more than 2 species of organisms, and associated with one of the following symptoms: fever >38°C, dysuria or suprapubic tenderness [19]. Patients were considered to have a pulmonary bacterial infection if they had dyspnea or cough and a new radiographic infiltrate for which a nonbacterial or noninfectious complication was unlikely according to the clinical circumstances. Bacteremia/sepsis was defined when blood cultures were positive and clinical signs or symptoms of infection were present, but without any other recognized cause. Cellulitis diagnosis was based on clinical features of infection such as fever or chills and the affected skin appears swollen, red, warm, and tender.

Diagnosis of variceal bleeding was made when patients presented with symptoms of upper GI bleeding (hematemesis, melena stools, or both) and bleeding varices confirmed by upper GI endoscopy.

Hepatocellular carcinoma was diagnosed based on any one of the following; clinical context (weight loss, hard nodular hepatomegaly or presence of chronic liver disease or cirrhosis) or positive serological markers of HBV or HCV infections and alpha-fetoprotein (AFP) levels >165.2 IU/ML, presence of a liver mass with characteristic HCC imaging changes on computed tomography (CT) or magnetic resonance imaging (MRI), alpha-fetoprotein (AFP) levels >165.2 IU/ML and liver mass on ultrasonography (USG) or histopathological confirmation of HCC [20, 21].

Risk factors/etiological spectrums of liver cirrhosis were defined as follows:

Chronic HBV was defined as a positive HBsAg test result and chronic HCV was defined as a positive anti-HCV antibodies test result.

Alcoholic etiology was made when the patient’s declared alcohol consumption was more than 14 units per week when measurable or local alcohol beverage consumption was three times per week in the past five years and correlated with biological abnormalities related to alcohol consumption [22].

Hepatic schistosomiasis was defined as the presence of Schistosoma mansoni ova in stool or previous history of Schistosoma mansoni and/or Ultrasound finding of hepatic schistosomiasis features such as Periportal fibrosis.

Nonalcoholic fatty liver disease (NAFLD) etiology was made when patients had risk factors for NAFLD and its progression and other causes of liver cirrhosis had been excluded.

Unidentified etiology referred to liver cirrhosis patients in whom no etiology was found.

Mortality was assessed at the index hospitalization. In-hospital mortality rate was defined as death occurring in the cohorts at the time of their index hospitalization. These included those who had compensated cirrhosis.

### Ethical approval and informed consent

Institutional Ethical and Review Committee of the St. Dominic Hospital approved this study. This study was conducted following the Helsinki Declaration on Human Experimentation, Sixth Revision (October 2008). The nature of the study was fully explained to potential participants. The patients were told that participation in the study was totally voluntary, and that abstaining would not affect their immediate or subsequent medical care at the hospital. Participants who decided to be part were asked to sign an informed consent form. For those patients with hepatic encephalopathy, written consent was obtained from caregivers.

### Statistical analysis

The data obtained was analyzed using the statistical package for social sciences (IBM SPSS, version 23) statistical software. Descriptive statistics were undertaken for all the variables and data presented in appropriate tables. The causes of liver cirrhosis and the reasons for admission were determined. Further analysis was done to determine if there were any associations between, survival or non-survival and the clinical or laboratory parameters. Chi-square was used to determine the level of association between two categorical variables. A binary logistic regression analysis was conducted for clinical, laboratory parameters, and prognostic scores (CPS, MELDNa) to determine if any of them were a predictor of death. For all analyses, a p-value of < 0.05 was considered statistically significant.

## Results

One hundred and eighty-six patients (186) with a median age of 46 years were included in the study, comprising of 124 (66.7%) males with a male to female ratio of 2:1. HBV was the main etiology of liver cirrhosis (38.7%) followed closely by alcohol consumption (38.**3**%). In 14(7.5%) of the patients, the causes of their liver cirrhosis were not identified. No significant differences were found in the distribution of age, sex, and etiology among survivors and non-survivors (Table 1).

**Table 1:** Sociodemographic characteristics and causes of liver cirrhosis in relation to their association with in-hospital survival.

Characteristics	Total	Survivors (n = 108)	Non-survivors (n = 78)	p-value
**Age, YRS,(IQR)**	46(20–78)	46.82(33–59.7)	47.5(34.5–60.5)	0.74
**Gender**				0.21
Male	124(66.7%)	76(40.9%)	48(25.8%)	
Female	62(33.3%)	32(17.2%)	30(16.1%)	
**Etiology**				0.17
Alcohol	71(38.2%)	39(54.9%)	32(45.1%)	0.50
Alcohol/HBV	9(4.8%)	5(55.6%)	4(44.4%)	0.88
Alcohol/HBV/HCV	1(0.5%)	0(0.0%)	1(100.0%)	0.24
HBV	72(38.7%)	45(62.5%)	27(37.5%)	0.47
HBV/HCV	2(1.1%)	0(0.0%)	2(100.0%)	0.10
HCV	6(3.2%)	1(16.7%)	5(83.3%)	**0.04**
HCV/Alcohol	2(1.1%)	1(50.0%)	1(50.0%)	0.82
NAFLD	5(2.7%)	3(60.0%)	2(40.0%)	0.93
Schistosomiasis	4(2.2%)	4(100.0%)	0 (0.0%)	0.05
Unknown	14(7.5%)	10(71.4%)	4(28.6%)	0.53

HBV–Hepatitis B virus; HCV–Hepatitis C virus; NAFLD–Nonalcoholic fatty liver disease.

The top five causes of hospitalization in the cohort were hepatic encephalopathy (32.8%), ascites (20.4%), hepatocellular carcinoma (19.9%) and infections (18.8%). With regards to hepatic encephalopathy, a little over one fifth (22.9%) of them presented initially with encephalopathy whereas encephalopathy occurred in 77.1% of those who died. Ascites the second commonest cause of admission occurred in 97.4% of survivors compared to 2.6% of non-survivors. HCC was more frequent in survivors than those who died. Infective causes contributed to about 55.6% in survivors and 54.4% in those who died. Pneumonia was also more frequent in survivors (60%) than in non survivors (40%). Other reasons for admission included variceal bleeding 8(4.3%), dehydration from gastroenteritis 5(2.7%), and for further workup in 2(1.1%). The in-hospital mortality rate was 41.9% (78/186). In-hospital mortality was significantly higher in participants who were admitted with hepatic encephalopathy (p < 0.001) and those with sepsis (p—0.02), compared with those who were admitted for other reasons. However, those admitted with ascites had a higher chance of survival compared with participants with other complications (p < 0.001) (Table 2).

**Table 2:** Reasons for admissions of the study participants in relation to their association with hospital outcome.

	Total(n = 186)	Survivors (n = 108	Non-survivors (n = 78)	p-value
Reasons for admissions				
Ascites	38(20.4%)	37/38(97.4%)	1/38(2.6%)	**<0.001**
Hepatic encephalopathy	61(32.8%)	14/61(22.9%)	47/61(77.1%)	**<0.001**
Gastroenteritis	5(2.7%)	5/5(100.0%)	0/5 (0.0%)	0.05
Hepatocellular carcinoma	37(19.9%)	25/37(67.6%)	12/37(32.4%)	0.19
Variceal bleeding	8(4.3%)	6/8(75.0%)	2/8(25.0%)	0.32
For further workup	2(2.0%)	2/2(100.0%)	0/2 (0.0%)	0.40
Infections	36 (18.8%)	20 /36(55.6%)	16/36 (54.4%)	0.43
*Malaria*	1(0.5%)	1/1(100.0%)	0/1 (0.0%)	0.40
*Pneumonia*	15(8.1%)	9/15(60.0%)	6/15(40.0%)	0.87
*Spontaneous bacterial peritonitis*	3(1.61%)	2/3(66.7%)	1/3(33.3%)	0.76
*Sepsis*	4(2.2%)	0/4(0.0%)	4/4(100.0%)	**0.02**
*Pulmonary tuberculosis*	1(0.5%)	0/1 (0.0%)	1/1(100.0%)	0.24
*Urinary tract infection*	8(4.3%)	6/8(75.0%)	2/8(25.0%)	0.32
*Cellulitis*	3(1.6%)	1/3(33.3%)	2/3(66.6%)	0.38

Baseline clinical features significantly associated with death were the presence of jaundice and weight loss whereas their admission laboratory parameters associated with mortality were high bilirubin (mainly conjugated), international normalized ratio (INR), creatinine, blood urea nitrogen (BUN), CPS, MELD-Na, and low sodium (Tables 3 and 4). In the logistic regression model, the independent predictors of in-hospital mortality were the presence of encephalopathy (OR: 4.499; CI: 1.598–12.665; p- 0.004), MELDNa (OR:1.73; CI: 1.366–2.192; p- <0.001), NR (OR: 0.282, CI:0.102–0.783, p-0.015), sodium (OR: 1.162, CI: 1.019–1.325, p-0.025) and BUN (OR: 1.276 CI: 1.066–1.528; p– 0.008) (Table 5).

**Table 3:** Clinical features of cirrhotic patients in relation to their association with in-hospital survival.

	Total (n = 186)	Survivors (n = 108	Non- survivors (n = 78)	p-value
Ascites				0.241
Moderate	55(29.6%)	27/55(49.1%)	28/55(50.9%)	
Mild	7(3.8%)	3/7(42.9%)	4/7(57.1%)	
Severe	91(48.9%)	59/91(64.8%)	32/91(35.2%)	
No ascites	33(17.7%)	19/33(57.6%)	14/33(42.4%)	
Pedal oedema	98(52.7%)	62/98(63.3%)	36/98(36.7%)	0.139
Jaundice	85(45.7%)	37/85(43.5%)	48/85(56.5%)	**<0.001**
Fever	33(17.7%)	14/33(42.4%)	19/33(57.6%)	0.053
Chills	21(11.3%)	9/21(42.9%)	12/21(57.1%)	0.161
Abdominal pain	70(37.6%)	44/70(62.9%)	26/70(37.1%)	0.358
Weight loss	149(80.1%)	80/149(53.7%)	69/149(46.3%)	**0.016**
Hematemesis	24(12.9%)	15/24(62.5%)	924(37.5%)	0.666

**Table 4:** Laboratory parameters of cirrhotic patients in relation to their association with in-hospital survival.

Laboratory parameters	Total (n = 186)	Survivors (n = 108)	Non-survivors (n = 78)	p-value
**Haemoglobin(g/dl) Median (IQR)**	10.0(7.2–12.8)	10.3(8.1–12.1)	9.5(8.2–11.8)	0.44
**WBC (109/L) Median (IQR)**	14.8(-28.5–58.1)	7.1(4.6–9.1)	9.4(5.3–16.3)	0.72
**Platelet (10**^**9**^**/L) Median (IQR)**	166.5(43.8–289.2)	143.0(83.8–214.7)	146.0(65.8–244.3)	0.36
**Total Bil. (umol/l) Median (IQR)**	108.4(-35.8–252.6)	31.6(15.3–99.5)	73.6(28.0–213.3)	**0.005**
**Direct bil. (umol/l) Median (IQR)**	66.3(-23.3–155.9)	18.0(9.2–60.3)	49.4(18.7–120.9)	**0.003**
**Protein (g/l). Median (IQR)**	69.1(53.1–85.1)	71.0(60.0–79.0)	72.0(62.7–81.1)	0.205
**Albumin (g/l) Median (IQR)**	28.3(20.4–36.2)	28.0(23.0–34.0)	26.0(22.0–32.2)	0.96
**ALT (u/l) Median (IQR)**	65.7(14.8–116.6)	49.3(31.8–79.0)	52.4(33.4–92.8)	0.95
**AST (u/l) Median (IQR)**	124.3(16.1–232.5)	91.4(51.9–161.3)	99.7(54.0–149.8)	0.98
**ALP (u/l) Median (IQR)**	414.7(23–806.4)	265.0(161.7–432.0)	313.1(177.0–436.6)	0.97
**GGT (u/l) Median (IQR)**	267.2(0.3–534.1)	149(78.0–324.0)	143.1(85.3–309.2)	0.62
**INR Median (IQR)**	2.4(1.5–3.3)	2.0(1.6–2.5)	2.5(1.9–3.2)	**<0.001**
**CPS Median (IQR)**	10.3(7.8–12.8)	10.0(8.0–11.0)	11.5(9.0–14.0)	**<0.001**
**MELD NA Median (IQR)**	25.45(17.2–33.8)	22.0(17.0–27.0)	29.0(23.8–35.3)	**<0.001**
**Sodium (mmol/l) Median (IQR)**	132.6(125.7–139.5)	134.0(130.0–138.0)	132(126.3–136.0)	**<0.001**
**Potassium (mmol/l) Median (IQR)**	4.1(3.3–4.9)	4.0(3.7–4.3)	4.0(3.6–4.6)	0.56
**Creatinine (umol/l) Median (IQR)**	127.4(12.1–242.7)	78.0(58.8–111.1)	115.5(63.8–213.2)	**<0.001**
**BUN (umol/l) Median (IQR)**	8.7(-0.1–17.5)	5.0(3.2–7.8)	7.7(4.2–14.6)	**<0.001**

ALT- Alanine aminotransferase; AST-Aspartate aminotransferase; ALP- Alkaline phosphatase; CPS- Child-Pugh score; GGT- Gamma-glutamyl transferase; INR-International Normalized Ratio; WBC-White Blood Cell Count; BUN-Blood Urea Nitrogen; MELDNa–Model for end-stage liver disease sodium; Bil–Bilirubin; IQR-Interquartile range.

**Table 5:** Logistic regression model of patients hospitalized with liver cirrhosis in relation to clinical findings as predictors of survival.

Variables	B	S.E.	Wald	p-value	Odds ratio	95% C.I.for EXP(B)	
						Lower	Upper
Total bilirubin (umol/l)	-0.02	0.015	1.69	0.194	0.98	0.951	1.01
INR	-1.263	0.52	5.91	**0.015**	0.282	0.102	0.783
CPs	-1.43	0.747	3.667	0.056	0.239	0.055	1.034
MELDNa	0.548	0.121	20.647	**0.001**	1.73	1.366	2.192
Sodium (mmol/l)	0.15	0.067	4.992	**0.025**	1.162	1.019	1.325
Creatinine (mmol/l)	0.005	0.004	1.340	0.247	1.005	0.997	1.014
BUN	0.244	0.092	7.082	**0.008**	1.276	1.066	1.528
Jaundice	0.443	0.859	0.266	0.606	1.557	0.289	8.382
Hepatic encephalopathy	1.504	0.528	8.109	**0.004**	4.499	1.598	12.665
Weight Loss	-0.973	0.547	3.162	0.075	0.378	0.129	1.105
Constant	2.367	6.657	0.126	0.722	10.661		

INR-International Normalized Ratio; BUN-Blood Urea Nitrogen; MELDNa–Model for end-stage liver disease sodium; Bil–Bilirubin; CPS- Child-Pugh score.

## Discussion

Liver disease is one of the most common chronic diseases in Ghana. Despite the high burden of liver cirrhosis in Ghana, data on this disease is lacking. This study aimed to determine the sociodemographic characteristics, reasons for admission, and in-hospital mortality of patients with cirrhosis of the liver seen at a district hospital in Ghana. The median age of hospital admissions from liver cirrhosis in this study was 46 years. This age group is the most socioeconomically active who offer support to their families and contribute immensely to the general economy. The high level of liver cirrhosis in this group is expected to impose a substantial unintended burden on the families and the economy through loss of productivity, benefit payments, and taxation. A similar age group has been reported from comparable studies in this country [23, 24] and other parts of Africa [22]. However, the median age in other studies conducted in western countries was higher than that of this study [25, 26]. Disparities in the median age in different study sites are likely to be related to dissimilarities in the etiologies and the frequency of chronic hepatitis B virus infection in different countries, the period of acquisition of the viral infection by the patients also influence the age at which cirrhosis of the liver develops. The male predominance of 2:1 reported in this study parallels that reported by Achinge et al. in Nigeria [27]. Other reports from studies conducted in sub-Saharan Africa and other western countries have shown comparable male predominance [7, 26]. A possible reason for this is that men are involved in events that exposes them to risk factors that causes liver cirrhosis such as intravenous drug abuse and alcoholism. Moreover, the socioeconomic factors affecting the health-seeking behavior of persons in many developing countries may be partly responsible. Many women are economically constrained and often must obtain permission and support from their spouses to seek medical care. Men are therefore more likely to seek care for their medical illnesses than their wives. Another school of thought is that men do not attend clinic unless it becomes critical.

Chronic hepatitis B was recognized as the main etiology of liver cirrhosis with HCV contributing less. This is compatible with the high HBV (13%) prevalence and a comparatively low prevalence of HCV (3.0%) in Ghana [6, 28]. This finding is comparable with other reports in literature from Africa and other hepatitis B endemic countries [22, 29]. Excessive alcohol intake was the second commonest cause of liver cirrhosis in the current study which implies that alcohol is a significant etiology of liver cirrhosis in patients seeking care at SDH, constituting 38.2% of all causes of liver cirrhosis. This is higher than 23.1% and 32.9% reported from previous studies conducted in a teaching hospital in Accra, Ghana [23, 30]. A study by Terefe Tesfaye et al. in Ethiopia [7], found HBV as the major cause of liver cirrhosis while in Sudan [31] alcohol abuse was the commonest cause. In Australia [32], alcohol was the commonest cause and in Greece [33] HCV was the major cause. The causes of liver cirrhosis are the same globally but the proportions of the individual etiologies differ from one country to another and even in the same country from one area to another. Though NAFLD is gradually emerging as an important cause of liver cirrhosis globally, this was the etiology in only 2.7% of our cohort. The reason may be due to the growing rate of obesity in the developed countries and Asia compared to African countries [34] or probably because of under diagnosis of NAFLD in Ghana.

Hepatic encephalopathy (32.8%), ascites (20.4%), hepatocellular carcinoma (19.9%), and infections (18.8%) were prevalent in a large proportion of hospitalized patients. These are similar to other studies that have been conducted on this subject but in variable proportions [7, 35]. Early identification of the following complications and their precipitating factors can help reduce the rate of admissions or death in these patients. In-hospital mortality in this study was 41.9%. This is similar to the in-hospital mortality rate of 41.0% reported in a study from Ethiopia [29] and 40% reported by de Sausmarez et al. [25] in a study conducted in the UK. However, a lower in-hospital mortality rate of 23.5% was reported by Zubieta-Rodríguez et al. in Columbia, and 28.4% in-hospital mortality reported by Tesfaye et al. [7, 9], in another Ethiopian study. However, a higher mortality rate of 53.3% and 48% were reported by Vaz et al. [36] and Cavallazzi et al respectively [26]. These differences could be due to the variations in the patient’s baseline characteristics and study settings. These characteristics include disease stage, associated comorbidities, complications of liver cirrhosis leading to admission, the clinical setting as well as the clinical status upon admission. For instance, the present study included participants admitted to the medical wards only, while others were conducted among patients admitted to both intensive care units and the internal medicine ward. Furthermore, late presentation, unavailability of definite therapies and advanced hepatology centers as pertains in Ghana and other African countries could have contributed to these disparities observed.

In this study, the group of patients who died had the highest values of creatinine, BUN, bilirubin, INR, CPS, MELDNa, and the lowest values of sodium, compared with the group of survivors. This implies that patients who died had more advanced liver disease and multiorgan dysfunction, similar to that of other studies published on this subject [37].

Various studies have identified certain laboratory variables as independent risk factors for mortality in cirrhosis and these include white cell count, creatinine, albumin, bilirubin, prolonged INR, and infections [7, 8, 35, 37–39]. Mortality was highest in patients admitted with hepatic encephalopathy, sepsis, jaundice, and weight loss. Other studies have also identified hepatic encephalopathy and sepsis as predictors of death in patients admitted with liver cirrhosis [7, 39]. The presence of jaundice and weight loss in cirrhotic patients reflects the deterioration of the underlying liver condition with death rates being higher in patients with decompensated liver cirrhosis. However, hepatic encephalopathy, high MELDNa, BUN, INR and low sodium that were independent predictor of poor outcome on logistic regression analysis. Those admitted with ascites had a higher chance of survival compared with those admitted with other complications. The better survival noticed in the ascites group in this study could be attributed to the fact that majority of the patients admitted with ascites were for therapeutic abdominal paracentesis and had no other major complications.

This study had some limitations. Firstly, it was difficult to identify the underlying complications that led to death because of the overlapping complications of cirrhosis. Secondly, the diagnosis of liver cirrhosis in this study was based mainly on clinical, laboratory, and radiologic examinations. This method of diagnosis without any histologic basis might have missed patients with early or compensated cirrhosis.

## Conclusion

The common causes of liver cirrhosis admitted at St. Dominic hospital were chronic HBV infection and excess alcohol consumption. In-hospital mortality of patients with liver cirrhosis was high and hepatic encephalopathy being the leading cause of death. An admission diagnosis of hepatic encephalopathy was associated with a poorer outcome. High MELDNa, INR, BUN and low sodium were scores and laboratory parameters that were independent predictors of death. The adaptation of public health measures directed towards the prevention of liver cirrhosis and early diagnosis of this disease is necessary to prevent the development of complications and improve outcomes in cirrhotic patients. Timely diagnosis and adequate management of hepatic encephalopathy are also necessary. A time- to- event analysis from first time of hospitalization to death should be considered in a future study.

## Supporting information

S1 Dataset Study questionnaire. (DOCX)

S2 Dataset Complications requiring hospital admission and in-hospital mortality of liver cirrhosis. (XLSX)

S3 Dataset STROBE statement, liver cirrhosis. (DOCX)
