# Supplementary material for: Sociodemographic characteristics, complications requiring hospital admission and causes of in-hospital death in patients with liver cirrhosis admitted at a district hospital in Ghana
Source: PLoS One. 2021 Jun 24;16(6):e0253759. doi: 10.1371/journal.pone.0253759 (PMC8224881; doi:10.1371/journal.pone.0253759)
Supplement: S1 Dataset — (DOCX) [file pone.0253759.s001.docx]

**Sociodemographic characteristics, complications requiring hospital admission and causes of in-hospital death in patients with liver cirrhosis admitted at a district hospital in Ghana.**

**Questionnaire**

You will be asked some questions about yourself as well as the circumstances surrounding your current condition. I assure you that any information you give us will be treated as confidential.

Thank you for your cooperation.

**PERSONAL AND DEMOGRAPHIC DATA**

**1. Folder No…………………. Identification No……………………..**

**2. Name (Initials)……………………………..**

3. Age...............

4. Gender

a. male b. Female

5. Ethnicity

a. Akan b. Ga/Adangbe c. Ewe d.Hausa e.others, specify........

6. Highest level of education

a. none b. primary c. middle school/JHS d. Senior High

e. Tertiary f. Post Graduate

7. Religion

a. Christian b. Muslim c. others, please specify................

8. Marital Status

a. married b. single c. divorced d. separated e. others, specify....

9. Employment status

a. Public b. Private c. Self-employed d. unemployed

e. Others, please specify

10. Occupation

Please specify................................................

**Clinical presentation**

1. Ascites
2. Absent b. Mild c. Moderate d. Severe e. On Diuretics
3. Hepatic encephalopathy
4. Absent b. Stage 1 c. Stage II d. Stage III e. Stage IV
5. Jaundice
6. Present b. Absent
7. Haematemesis
8. Present b. Absent
9. Mealena stools
10. Present b. Absent
11. Weight loss
12. Present b. Absent
13. Fatigue
14. Present b. Absent
15. Others, Please specify ………………………………………………………………………………………………………………………………………………………………………………………………………………………………………………
16. Alcohol consumption a. Yes b. No

If yes

1. Duration (years)………………………………………..
2. Amount (estimated units/day)…………………………………………
3. Type or Bland……………………………………………….

**Laboratory and Radiological investigations**

1. Full Blood Count
2. Platelet Count………………………….(N/mm^3^) b. Hb…………………..g/l c. WBC………………………
3. Liver Function Test

a. AST……………………..(U/L) b. ALT…………………………(u/l)

c. Serum albumin…………………..(g/l) d. Total protein………….(g/l)

e. Total bilirubin……………(umol/l) f. D. bilirubin……………(umo/l)

g. INR……………………… h. GGT………………………….(u/l)

i. AKL………………(U/l) j. AST/ALT ratio………………

Baseline BUE and Cr

1. Na………………. b. K……………….. c.Cr…………………. d.BUN…………………………

Subsequent/Follow up BUE and Cr

1. Na……………… b. K……………… c. Cr…………………….. d. BUN…………………………..

3. Ascitic fluid analysis

1. Albumin………………….. b. Total protein…………………… c. Total cell count……………
2. Neutrophils …………….. e. Lymphocytes………………… f. Erythrocytes…………………

4. HBSAg a. Positive b. Negative

5. HCV Ab a. Positive b. Negative

6. Retroviral test a. Positive b. Negative

7. Urine routine examination

1. Proteins ……………………….. b. leucocytes…………….. c. erythrocytes
2. Pus cells ……………………….
3. Other abnormalities please specify…………………………………………………………………………………..

Urine C/S……………………………………………….

8. Chest X-ray

9. Other test when necessary

i. Antinuclear antibody vi. Serum IgG

ii. antismooth muscles antibody vii. alpha 1 antitrypsin level

iii. antimitochondrial antibody viii. Serum Iron

iv anti liver/kidney microsomal 1 antibodies ix. Total iron binding capacity

1. anti – liver cytosol 1 antibodies x. Serum caeruloplasmin
2. Fasting blood sugar xii. Lipid profile xiii. CXR…………………………

9. Esophageal varices a. Absent b. Small varices c. large Varices

10. Child Pugh score………………………………………………………………………………..

11. MELD-Na+…………………………………………………………………………………………..

12. Serum- Ascitic albumin gradient (SAAG)………………………………………………………………………

13. Outcome of admission ……… a. Discharge b. Death

14. Immediate cause of death ………………………………………………………………….

**Child-Turcotte-Pugh Scoring System for Cirrhosis**

| **Clinical Variable** | **1 Point** | **2 Points** | **3 Points** |
| --- | --- | --- | --- |
| Encephalopathy | None | Grade 1-2 | Grade 3-4 |
| Ascites | Absent | Slight | Moderate or large |
| Bilirubin (mg/dL) | < 2 | 2-3 | >3 |
|  |  |  |  |
| Albumin (g/dL) | >3.5 | 2.8-3.5 | < 2.8 |
| Prothrombin time(seconds prolonged or INR) | < 4 s or INR < 1.7 | 4-6 s or INR 1.7-2.3 | >6 s or INR >2.3 |
|  |  |  |  |

**Grading of Ascites**

Grade 1 (mild). Ascites is only detectable by ultrasound examination.

Grade 2 (moderate). Ascites causing moderate symmetrical distension of the abdomen.

Grade 3 (severe). Ascites causing marked abdominal distension.

**West Haven grading system of hepatic encephalopathy**

Grade 0 – Minimal hepatic encephalopathy lack of detectable changes in personality or behaviour; minimal changes in memory, concentration, intellectual function, and coordination; asterixis is absent.

Grade 1 – Trivial lack of awareness; shortened attention span; impaired addition or subtraction; hypersomnia, insomnia, or inversion of sleep pattern; euphoria, depression, or irritability; mild confusion; slowing of ability to perform mental tasks.

Grade 2 – lethargy or apathy; disorientation; inappropriate behaviour; slurred speech; obvious asterisxis; drowsiness, lethargy, gross deficits in ability to perform mental tasks, obvious personality changes, inappropriate behaviour, and intermittent disorientation, usually regarding time.

Grade 3 – Somnolent but can be aroused; unable to perform mental tasks; disorientation about time and place; marked confusion; amnesia; occasional fits or rage; present but incomprehensible speech.

Grade 4 – Coma with or without response to painful stimuli.
