# Supplementary material for: Sociodemographic characteristics, complications requiring hospital admission and causes of in-hospital death in patients with liver cirrhosis admitted at a district hospital in Ghana
Source: PLoS One. 2021 Jun 24;16(6):e0253759. doi: 10.1371/journal.pone.0253759 (PMC8224881; doi:10.1371/journal.pone.0253759)
Supplement: S3 Dataset — (DOCX) [file pone.0253759.s003.docx]

STROBE Statement—Checklist of items that should be included in reports of ***cohort studies***

|  | Item No | Recommendation |
| --- | --- | --- |
| **Title and abstract** | 1 | (*a*) Indicate the study’s design with a commonly used term in the title or the abstract  **Sociodemographic characteristics, Complications Requiring Hospital Admission and Causes of In-Hospital Death in Patients with liver Cirrhosis Admitted at a District Hospital in Ghana: A prospective cohort study.** |
|  |  | (*b*) Provide in the abstract an informative and balanced summary of what was done and what was found  **Abstract**  **Background:** Chronic liver diseases including liver cirrhosis are a major cause of morbidity and mortality globally. Despite the high burden of liver cirrhosis in Ghana, data on this disease is lacking.  **Objective:** To determine the sociodemographic characteristics, reasons for admission, and in-hospital mortality of patients with cirrhosis of the liver seen at a district hospital in Ghana.  **Design:** Prospective Cohort Study  **Setting:** St. Dominic Hospital, Ghana  **Patients:** One hundred and eighty-six (186) patients admitted on the medical wards in St. Dominic hospital with liver cirrhosis from 1st January 2018 to 24th June 2020.  **Methodology:** The patient's demographic and clinical features were documented using a standardized questionnaire. Diagnostic biochemical and haematological tests as well as abdominal ultrasound scans were performed for all patients. They were followed up until death or discharge from hospital.  **Results:** One hundred and eighty-six patients (186) with a median age of 46 years were included in the study. HBV was the main etiology of liver cirrhosis (38.7%) followed closely by alcohol consumption (38.3%). In-hospital mortality was 41.3% and the most frequent cause of death was hepatic encephalopathy (68.4%). The following were associated with death; Jaundice, weight loss, elevated bilirubin, international normalized ratio (INR), creatinine, blood urea nitrogen, Child-Pugh score, model for end-stage liver disease sodium score, and low sodium. However, hepatic encephalopathy, MELDNa, INR and BUN were independent predictors of in-hospital mortality on logistic regression analysis.  **Conclusions:** In-hospital mortality in cirrhotic patients was high with the leading cause of death being hepatic encephalopathy. Timely diagnosis and adequate management of hepatic encephalopathy are necessary to prevent death from liver cirrhosis. |
| Introduction | | |
| Background/rationale | 2 | Explain the scientific background and rationale for the investigation being reported  Chronic liver diseases (CLD) are a major cause of morbidity and mortality globally including Ghana [1]. Cirrhosis is the end-stage of all chronic liver diseases and is characterized by progressive fibrosis, scarring, and formation of regenerative nodules resulting in distortion of the normal liver architecture [2]. The burden of cirrhosis differs considerably across various geographical locations and according to sex, race, ethnicity, and socioeconomic level. There has also been a significant variation in the burden of cirrhosis over time. Mortality from liver cirrhosis increased worldwide from less than 899,000 in 1990 to over 1.32 million in 2017. In 2017, this constituted 2.4% of all deaths compared with 1.9% in 1990 [3]. Sub-Saharan Africa had the highest age-standardized mortality rate (32.2 deaths per population) according to the global burden of disease super-regions for 2017 [3]. From the global burden of disease study 2010, liver cirrhosis was the 13^th^ commonest cause of premature death in Ghana [4]. Chronic hepatitis B and C, excessive alcohol consumption and non-alcoholic fatty liver disease have been found to be the commonest causes of liver cirrhosis globally [5]. Chronic HBV infection is endemic in Ghana [6]. Despite being a worldwide health challenge with a major public health and economic burden, data assessing in-hospital mortality and morbidity due to cirrhosis are sparse, especially in many regions such as Africa [3]. In-hospital death in patients with liver cirrhosis and predictors of mortality are variable in various studies reported in literature. In a study conducted in Ethiopia by Terefe Tesfaye et al. [7], in-hospital mortality of cirrhotic patients was found to be 28.4% with hepatic encephalopathy and high bilirubin as predictors of death. This study, however, was limited by the small sample size and inability to perform advanced images like CT scan and MRI. It also did not have access to endoscopy to diagnose oesophageal varices. A similar retrospective study conducted in Morocco by Charif et al. [8], also found in-patients death of liver cirrhosis to be 8.7% with hepatic encephalopathy, hyponatremia, high creatinine, and leucocytes as predicting factors of death. Zubieta-Rodríguez et al. [9], reported 23.5% in-hospital mortality with high MELD score, leucocyte count and low albumin as independent factors related to death in Columbia and in-hospital mortality rate in a study conducted in Saudi Arabia was 35% with advanced age and MELD scores as predictors of death [10].  Data from Ghana on liver cirrhosis is scarce and therefore the gravity of liver cirrhosis is likely to be underestimated. Few studies have been carried out on liver diseases burden in Ghana. One of such studies reported cirrhosis as the commonest cause of death attributable to liver diseases in the country [11]. Moreover, existing studies are old and largely retrospective. There is a dearth of knowledge about in-hospital mortality of liver cirrhosis patients and the predictors of death in Ghana. This study aimed to assess the baseline characteristics, causes of hospital admissions, in-hospital mortality and predictors amongst patients with liver cirrhosis in a district hospital in Ghana. |
| Objectives | 3 | State specific objectives, including any prespecified hypotheses  **SPECIFIC OBJECTIVES**: The primary outcome of interest was the cause of in-hospital mortality and the secondary outcome was predictors of in-hospital mortality.  1. To determine the prevalence of in-hospital mortality in patients with liver cirrhosis.  2. To determine predictors of in-hospital mortality in patient with liver cirrhosis  3. To determine sociodemographic characteristics of patients with liver cirrhosis.  **HYPOTHESIS:** |
| Methods | | |
| Study design | 4 | Present key elements of study design early in the paper  This was a descriptive, observational prospective hospital-based study. The study population comprised adult patients with liver cirrhosis admitted to the medical unit of SDH during the study period who met the inclusion criteria. A questionnaire was administered to obtain information on socio-demographic data as well as relevant clinical history such as alcohol use. All patients underwent a thorough physical examination, and any stigmata of liver cirrhosis were documented. Further information collected included reasons for admission and the presence of cirrhosis-related compli­cations such as ascites, variceal bleeding, spontaneous bacterial peritonitis (SBP) and other infections, hepatic encephalopathy and acute kidney injury including hepatorenal syndrome (HRS). Biochemical, haematological, serological and imaging studies were done for all patients  The data obtained was analyzed using the statistical package for social sciences (IBM SPSS, version 23) statistical software. |
| Setting | 5 | Describe the setting, locations, and relevant dates, including periods of recruitment, exposure, follow-up, and data collection  The study was carried out at the Department of Medicine, St. Dominic Hospital (SDH) in Akwatia, Ghana from 1st January 2018 to 24th June 2020. SDH is a 339-bed district hospital located in the Denkyembour district, Akwatia in the Eastern region of Ghana and serves as the main referral center for surrounding district hospitals. |
| Participants | 6 | 1. Give the eligibility criteria, and the sources and methods of selection of participants. Describe methods of follow-up   Patients with a diagnosis of liver cirrhosis (compensated or decompensated) who were 18 years and above and admitted for the first time during the study period were included.  Patients with a previous history of HCC **and those** that were re-admitted within the study period.  Patients were recruited into the study on their index admission to SDH after meeting the inclusion criteria. Patients were followed up from this index admission until death or discharge. In-hospital mortality rate was defined as death occurring in the cohorts at the time of their index hospitalization |
|  |  | (*b*) For matched studies, give matching criteria and number of exposed and unexposed |
| Variables | 7 | Clearly define all outcomes, exposures, predictors, potential confounders, and effect modifiers. Give diagnostic criteria, if applicable.  Only patients with colorectal cancer cases were included. All other large bowl conditions were excluded. In the statistical analysis, factors such as age and presence of comorbidities were taking into consideration as potential confounding factors.  Outcome; Dead or alive (Survival)  Exposure; Liver cirrhosis  Predictors; Socio-demographic factors, Clinical features and laboratory parameters  Potential confounders: Age, sex, aetiology of liver cirrhosis  Effect modifiers; treatment administered to the patients for complications leading to their admission and the underlying cause of liver cirrhosis. |
| Data sources/ measurement | 8* | For each variable of interest, give sources of data and details of methods of assessment (measurement). Describe comparability of assessment methods if there is more than one group.  Mortality was assessed at the index hospitalization. In-hospital mortality rate was defined as death occurring in the cohorts at the time of their index hospitalization. All patients were follow-up whiles on admission until death or discharged.  All other information in relation to socio-demographic characteristics, clinical features and laboratory parameters were obtained from the patients/medical records within 24 hours of admission. |
| Bias | 9 | Describe any efforts to address potential sources of bias  Types of possible bias; Information bias.  Because of the short follow up of the participants and the fact that they were all in-patients, we ensured all needed data were collected. |
| Study size | 10 | Explain how the study size was arrived at  The sample size was determined using the Cochran formula for sample size calculation. With an estimated prevalence of 10.7% for in-hospital mortality of liver cirrhosis [12], a Z-score at 95% confidence level (1.96), and a level of significance of 0.05, the minimum sample size was calculated to be 148. Due to the high attrition rate for prospective studies, 186 participants were consecutively recruited after meeting study criteria and giving informed consent. |
| Quantitative variables | 11 | Explain how quantitative variables were handled in the analyses. If applicable, describe which groupings were chosen and why  Quantitative variables were normalised and analysed using SPSS. |
| Statistical methods | 12 | Describe all statistical methods, including those used to control for confounding  The data obtained was analyzed using the statistical package for social sciences (IBM SPSS, version 23) statistical software. Descriptive statistics were undertaken for all the variables and data presented in appropriate tables. The causes of liver cirrhosis and the reasons for admission were determined. Further analysis was done to determine if there were any associations between, survival or non-survival and the clinical or laboratory parameters. Chi-square was used to determine the level of association between two categorical variables. A binary logistic regression analysis was conducted for clinical, laboratory parameters, and prognostic scores (CPS, MELDNa) to determine if any of them were a predictor of death. For all analyses, a p-value of < 0.05 was considered statistically significant. |
|  |  | (*b*) Describe any methods used to examine subgroups and interactions |
|  |  | (*c*) Explain how missing data were addressed  Patients with incomplete data were excluded from the study |
|  |  | (*d*) If applicable, explain how loss to follow-up was addressed |
|  |  | (*e*) Describe any sensitivity analyses |
| Results | | |
| Participants | 13* | (a) Report numbers of individuals at each stage of study—eg numbers potentially eligible, examined for eligibility, confirmed eligible, included in the study, completing follow-up, and analysed.  In all 186 patients were enrolled for this study. Among the patients, 108 were discharged, and 78 died. |
|  |  | (b) Give reasons for non-participation at each stage |
|  |  | (c) Consider use of a flow diagram |
| Descriptive data | 14* | (a) Give characteristics of study participants (eg demographic, clinical, social) and information on exposures and potential confounders  Demographic data of study participants include age, gender, and alcohol intake history. Clinical and laboratory parameters including clinical presentation of liver cirrhosis, liver function test, blood urea and creatinine, full blood count, hepatitis b and c status of the participants, MELDNa and Child-Pugh score. |
|  |  | (b) Indicate number of participants with missing data for each variable of interest  n/a |
|  |  | (c) Summarise follow-up time (eg, average and total amount)  The study period includes patients diagnosed with liver cirrhosis from1st January 2018 to 24th June 2020  The median length of hospital stay was 10.37 days. |
| Outcome data | 15* | Report numbers of outcome events or summary measures over time |
| Main results | 16 | (*a*) Give unadjusted estimates and, if applicable, confounder-adjusted estimates and their precision (eg, 95% confidence interval). Make clear which confounders were adjusted for and why they were included |
|  |  | (*b*) Report category boundaries when continuous variables were categorized |
|  |  | (*c*) If relevant, consider translating estimates of relative risk into absolute risk for a meaningful time period |
| Other analyses | 17 | Report other analyses done—eg analyses of subgroups and interactions, and sensitivity analyses |
| Discussion | | |
| Key results | 18 | Summarise key results with reference to study objectives  In this study, the median age was found to be 46 years. HBV was the main etiology of liver cirrhosis (38.7%) followed closely by alcohol consumption (38.3%). In-hospital mortality was 41.3% and the most frequent cause of death was hepatic encephalopathy (68.4%). The following were associated with death; Jaundice, weight loss, elevated bilirubin, international normalized ratio (INR), creatinine, blood urea nitrogen, Child-Pugh score, model for end-stage liver disease sodium score, and low sodium. However, hepatic encephalopathy, MELDNa, INR and BUN were independent predictors of in-hospital mortality on logistic regression analysis |
| Limitations | 19 | Discuss limitations of the study, considering sources of potential bias or imprecision. Discuss both direction and magnitude of any potential bias  Firstly, it was difficult to iden­tify the underlying complications that led to death because of the overlapping complications of cirrhosis. Secondly, the diagnosis of liver cirrhosis in this study was based mainly on clinical, laboratory, and radiologic examinations. This method of diagnosis without any histologic basis might have missed patients with early or compensated cirrhosis.  However, this is how cirrhosis is diagnosed at the study centre and almost all health facilities in Ghana. |
| Interpretation | 20 | Give a cautious overall interpretation of results considering objectives, limitations, multiplicity of analyses, results from similar studies, and other relevant evidence  Liver disease is one of the most common chronic diseases in Ghana. Despite the high burden of liver cirrhosis in Ghana, data on this disease is lacking. This study aimed to determine the sociodemographic characteristics, reasons for admission, and in-hospital mortality of patients with cirrhosis of the liver seen at a district hospital in Ghana. The median age of hospital admissions from liver cirrhosis in this study was 46 years. This age group is the most socioeconomically active who offer support to their families and contribute immensely to the general economy. The high level of liver cirrhosis in this group is expected to impose a substantial unintended burden on the families and the economy through loss of productivity, benefit payments, and taxation. A similar age group has been reported from comparable studies in this country [23, 24] and other parts of Africa [22]. However, the median age in other studies conducted in western countries was higher than that of this study [25, 26]. Disparities in the median age in different study sites are likely to be related to dissimilarities in the etiologies and the frequency of chronic hepatitis B virus infection in different countries, the period of acquisition of the viral infection by the patients also influence the age at which cirrhosis of the liver develops. The male predominance of 2:1 reported in this study parallels that reported by Achinge et al. in Nigeria [27]. Other reports from studies conducted in sub-Saharan Africa and other western countries have shown comparable male predominance [7, 26]. A possible reason for this is that men are involved in events that exposes them to risk factors that causes liver cirrhosis such as intravenous drug abuse and alcoholism. Moreover, the socioeconomic factors affecting the health-seeking behavior of persons in many developing countries may be partly responsible. Many women are economically constrained and often must obtain permission and support from their spouses to seek medical care. Men are therefore more likely to seek care for their medical illnesses than their wives. Another school of thought is that men do not attend clinic unless it becomes critical.  Chronic hepatitis B was recognized as the main etiology of liver cirrhosis with HCV contributing less. This is compatible with the high HBV (13%) prevalence and a comparatively low prevalence of HCV (3.0%) in Ghana [6, 28]. This finding is comparable with other reports in literature from Africa and other hepatitis B endemic countries [22, 29]. Excessive alcohol intake was the second commonest cause of liver cirrhosis in the current study which implies that alcohol is a significant etiology of liver cirrhosis in patients seeking care at SDH, constituting 38.2% of all causes of liver cirrhosis. This is higher than 23.1% and 32.9% reported from previous studies conducted in a teaching hospital in Accra, Ghana [23, 30]. A study by Terefe Tesfaye et al. in Ethiopia [7], found HBV as the major cause of liver cirrhosis while in Sudan [31] alcohol abuse was the commonest cause. In Australia, [32] alcohol was the commonest cause and in Greece [33] HCV was the major cause. The causes of liver cirrhosis are the same globally but the proportions of the individual etiologies differ from one country to another and even in the same country from one area to another. Though NAFLD is gradually emerging as an important cause of liver cirrhosis globally, this was the etiology in only 2.7% of our cohort. The reason may be due to the growing rate of obesity in the developed countries and Asia compared to African countries [34] or probably because of under diagnosis of NAFLD in Ghana.  Hepatic encephalopathy (32.8%), ascites (20.4%), hepatocellular carcinoma (19.9 %), and infections (18.8%) were prevalent in a large proportion of hospitalized patients. These are similar to other studies that have been conducted on this subject but in variable proportions. [7, 35]. Early identification of the following complications and their precipitating factors can help reduce the rate of admissions or death in these patients. In-hospital mortality in this study was 41.9%. This is similar to the in-hospital mortality rate of 41.0% reported in a study from Ethiopia [29] and 40% reported by de Sausmarez et al. [25] in a study conducted in the UK. However, a lower in-hospital mortality rate of 23.5% was reported by Zubieta-Rodríguez et al. [ in Columbia, and 28.4% in-hospital mortality reported by Tesfaye et al. [7, 9], in another Ethiopian study. However, a higher mortality rate of 53.3% and 48% were reported by Vaz et al. [36] and Cavallazzi et al respectively [26]. These differences could be due to the variations in the patient’s baseline characteristics and study settings. These characteristics include disease stage, associated comorbidities, complications of liver cirrhosis leading to admission, the clinical setting as well as the clinical status upon admission. For instance, the present study included participants admitted to the medical wards only, while others were conducted among patients admitted to both intensive care units and the internal medicine ward. Furthermore, late presentation, unavailability of definite therapies and advanced hepatology centers as pertains in Ghana and other African countries could have contributed to these disparities observed.  In this study, the group of patients who died had the highest values of creatinine, BUN, bilirubin, INR, CPS, MELDNa, and the lowest values of sodium, compared with the group of survivors. This implies that patients who died had more advanced liver disease and multiorgan dysfunction, similar to that of other studies published on this subject [37]. Various studies have identified certain laboratory variables as independent risk factors for mortality in cirrhosis and these include white cell count, creatinine, albumin, bilirubin, prolonged INR, and infections [7, 8, 35, 37-39]. Mortality was highest in patients admitted with hepatic encephalopathy, sepsis, jaundice, and weight loss. Other studies have also identified hepatic encephalopathy and sepsis as predictors of death in patients admitted with liver cirrhosis [7, 39]. The presence of jaundice and weight loss in cirrhotic patients reflects the deterioration of the underlying liver condition with death rates being higher in patients with decompensated liver cirrhosis. . However, hepatic encephalopathy, high MELDNa, BUN, INR and low sodium that were independent predictor of poor outcome on logistic regression analysis. Those admitted with ascites had a higher chance of survival compared with those admitted with other complications. The better survival noticed in the ascites group in this study could be attributed to the fact that majority of the patients admitted with ascites were for therapeutic abdominal paracentesis and had no other major complications |
| Generalisability | 21 | Discuss the generalisability (external validity) of the study results  Findings from this study cannot be generalised since only patients who were diagnosed and treated at one hospital (SDH) were included, hence this may not be a true reflection of the situation in the entire country. |
| Other information | | |
| Funding | 22 | Give the source of funding and the role of the funders for the present study and, if applicable, for the original study on which the present article is based  The authors declare that there is no conflict of interests regarding the publication of this paper. |

*Give information separately for exposed and unexposed groups.

**Note:** An Explanation and Elaboration article discusses each checklist item and gives methodological background and published examples of transparent reporting. The STROBE checklist is best used in conjunction with this article (freely available on the Web sites of PLoS Medicine at http://www.plosmedicine.org/, Annals of Internal Medicine at http://www.annals.org/, and Epidemiology at http://www.epidem.com/). Information on the STROBE Initiative is available at http://www.strobe-statement.org.
